# Pathologic and Radiographic Studies of Intrahepatic Metastasis Hepatocellular Carcinoma; The Role of Efferent Vessels

**DOI:** 10.1155/1996/75210

**Published:** 1996

**Authors:** Akihiro Toyosaka, Eizo Okamoto, Masao Mitsunobu, Takeshi Oriyama, Norio Nakao, Koui Miura

**Affiliations:** 1 First Department of Surgery Hyogo College of Medicine Nishinomiya Hyogo Japan; 2 Department of Radiology Hyogo College of Medicine Nishinomiya Hyogo Japan

## Abstract

The efferent vessel of hepatocellular carcinoma (HCC) and the mechanism and pathogenesis of the high frequency of intrahepatic metastasis in HCC has not yet been clarified. Three hundred ninety-three resected specimens of HCC were examined for tumor thrombosis in the portal vein and the hepatic vein: 231 tumors ≤5 cm in diameter were examined for the relationship between mode of tumor spread and tumor size. Efferent vessels in HCC were identified by direct injection of radiopaque material into the tumor in 23 resected liver specimens and by percutaneous infusion of radiopaque media into tumor nodules in 8 patients. The mode of tumor spread in HCC progressed from capsular invasion to extracapsular invasion, then to vascular invasion, and finally to intrahepatic metastasis. There was a strong statistical correlation between the presence of intrahepatic metastasis and portal vein thrombosis (p<0.05, R=0.998). Radiopaque material injected directly into 23 resected tumors entered only the portal vein in 17 tumors and into both the portal and hepatic veins in 6 tumors. In all 8 patients with unresectable lesions, radiopaque media injected percutaneously into tumor nodules flowed only into the portal vein. These findings suggest that intrahepatic invasion by HCC may occur through the portal vein as an efferent tumor vessel.


*HPB Surgery*, 1996, Vol. 10, pp. 97–104
Reprints available directly from the publisher
Photocopying permitted by license only

© 1996 OPA (Overseas Publishers Association)
Amsterdam B.V. Published in The Netherlands
by Harwood Academic Publishers
Printed in Malaysia

### Pathologic and Radiographic Studies of Intrahepatic Metastasis in Hepatocellular Carcinoma; The Role of Efferent Vessels

AKIHIRO TOYOSAKA[1], EIZO OKAMOTO[1], MASAO MITSUNOBU[1], TAKESHI ORIYAMA[1], NORIO NAKAO[2] and KOUI MIURA[2]

[1]First Department of Surgery and [2]Department of Radiology, Hyogo College of Medicine, Nishinomiya, Hyogo, Japan

*(Received 1 July 1995)*

The efferent vessel of hepatocellular carcinoma (HCC) and the mechanism and pathogenesis of the high frequency of intrahepatic metastasis in HCC has not yet been clarified. Three hundred ninety-three resected specimens of HCC were examined for tumor thrombosis in the portal vein and the hepatic vein: 231 tumors ≤5 cm in diameter were examined for the relationship between mode of tumor spread and tumor size. Efferent vessels in HCC were identified by direct injection of radiopaque material into the tumor in 23 resected liver specimens and by percutaneous infusion of radiopaque media into tumor nodules in 8 patients. The mode of tumor spread in HCC progressed from capsular invasion to extracapsular invasion, then to vascular invasion, and finally to intrahepatic metastasis. There was a strong statistical correlation between the presence of intrahepatic metastasis and portal vein thrombosis ($p<0.05$, $R=0.998$). Radiopaque material injected directly into 23 resected tumors entered only the portal vein in 17 tumors and into both the portal and hepatic veins in 6 tumors. In all 8 patients with unresectable lesions, radiopaque media injected percutaneously into tumor nodules flowed only into the portal vein. These findings suggest that intrahepatic invasion by HCC may occur through the portal vein as an efferent tumor vessel.

KEY WORDS: Hepatocellular carcinoma   intrahepatic metastasis   efferent vessel   portal vein thrombosis

### INTRODUCTION

The prognosis of hepatocellular carcinoma (HCC) has improved in recent years. This is the result of refined surgical techniques, early detection of small tumors by advances in imaging techniques and biochemical diagnosis, and the introduction of percutaneous arterial embolization and ethanol infusion therapy. [1–5] However, the prognosis of HCC remains relatively poor because of the high frequency of multiple hepatic tumors, although patients rarely have distant metastases at the time of surgery[1, 6, 7].

There are some cases of multicentric origin[7, 8], however, multiple lesions in HCC have been attributed to intrahepatic metastases[1, 5, 9–12] because HCC has a high frequency of tumor invasion into the portal vein system. The mechanism and pathogenesis of the high frequency of intrahepatic metastasis and portal invasion in HCC has not yet been clarified. To investigate the mechanism of intrahepatic metastasis and portal invasion, the pathological, radiological, and morphological studies were undergone to evaluate the efferent tumor vessels in HCC.

### MATERIALS AND METHODS

Four hundred and seventy-two patients with HCC underwent resection between April 1980 and December 1993 in our department; of those patients, 393 who underwent subsegmentectomy or larger resection were selected for entry into the study. The other patients who had smaller resection than subseg-

*Correspondence to*: Akihiro Toyosaka, M.D. First Department of Surgery, Hyogo College of Medicine, 1-1 Mukogawa-cho, Nishinomiya, Hyogo 663, Japan, Telephone number: 798-45-6582 Facsimile number: 798-45-6581

mentectomy, such as enucleation of the tumor or wedge resection of the tumor due to liver dysfunction were excluded from the study, because intrahepatic metastatic lesions could not be examined adequately.

The resected specimens were sliced at a thickness of about 1cm, and all of the slices were macroscopically examined for tumor thrombosis in the portal and hepatic veins. Two to three slices including the maximum cut-surface of the tumor were histologically examined over the total cut-surface. The frequency of capsular invasion, extracapsular invasion, vascular invasion, and intrahepatic metastasis were noted. Tissues were fixed in 10% buffered formalin and embedded in paraffin, processed routinely, and stained with hematoxylin and eosin. Selected sections were stained with periodic acid-Schiff (PAS), Masson trichrome, elastica-van Gieson, and silver impregnation.

Of the 393 total specimens, 231 showed tumors ≤5 cm in diameter. Of these, 23 (10%) were selected at random for radiographic evaluation. Radiopaque media was manually infused directly into the tumor immediately after hepatectomy using a 27 gauge needle with a low injection pressure and specimen radiographs were obtained. Specimens were then fixed and serially sectioned, and efferent tumor vessels were histopathologically evaluated. In 7 specimens, radiopaque media was infused into the portal vein, and the vasculature of the portal system around the tumor was evaluated in a similar fashion. In 3 specimens, molds of efferent tumor vessels were produced by infusing silicon rubber directly into the resected tumor.

In 8 patients with unresectable lesions, because of the advanced stage of the tumor or the advanced cirrhotic stage, radiopaque media was infused into the tumor by direct percutaneous puncture using an ultrasound guided puncture technique and efferent tumor vessels were observed. Subsequently percutaneous administration of chemotherapy with Adriamycin or percutaneous ethanol injection were conducted. The infusion of the radiopaque media into the tumor was approved by the Human Ethics Committee of the Hyogo College of Medicine, and written informed consent was obtained from each patient.

### *Statistical Analysis*

Differences in proportion between groups were made using chi square test or Fisher's exact test. The correlation between the presence of vascular invasion and intrahepatic metastasis was evaluated by correlation analysis. $P<0.05$ was regarded as significant.

### RESULTS

### *Histopathologic Findings*

The overall incidence of macroscopic intrahepatic metastasis was 205 of the 393 resected cases (52.1%). None had evidence of lung or bone metastasis at the time of surgery. Lymph node metastasis was also uncommon (1.6%) in the 393 cases.

When multiple intrahepatic tumors were present, the primary lesion was easily identified in all cases. Daughter nodules were generally smaller, peripheral to the primary tumor, and situated in the same portal branch area as the main tumor. Tumors tended to invade the portal vein and metastasize extensively even when the primary lesion was small and especially when it was located near the main trunk of the portal vein. Portal vein tumor thrombosis was observed in 73 cases (18.6%) macroscopically, while, tumor thrombosis of the hepatic vein was confirmed in only 12 cases (3.1%). Among the 12 cases, 11 also had extensive tumor thrombosis of the portal vein.

The pathology of HCC growth appeared to occur by expansion and compression of surrounding tissues. An abundant vascular network was noted in tumor capsules, and communicating branches between blood spaces in the tumor and this vascular network were observed in all cases. Serial sections showed that tumor cells were disseminated through these communicating branches and were also present within the vascular network of the capsule in most of the resected specimens. Tumor cells also were often noted within extracapsular blood vessels. Tumor cell infiltration to the intra- and extracapsular blood vessels was seen at many sites of all circumference (Figure 1).

The mode of tumor spread was histologically analyzed among 231 tumors ≤5 cm in diameter. Intracapsular invasion, which included isolated invasion of the vascular network of the capsule, was observed in 99.1% of cases. Extracapsular invasion was observed in 60.6% of HCC tumors <2.0 cm, in 76.3% of those 2.1 to 3.0 cm, and in 81.9% of those 3.1 to 5.0 cm in diameter. Invasion of pericapsular vessels (vascular invasion) was observed in 48.4% of HCC tumors <2.0 cm, in 59.1% of those 2.1 to 3.0 cm, and in 73.3% of those 3.1 to 5.0 cm in diameter. Thus, the larger the tumor size, significantly the higher was the frequency of extracapsular invasion, vascular invasion and intrahepatic metastasis. Metastasis to liver parenchyma was observed in 36.3% of HCC tumors <2.0 cm, in 49.4% of those 2.1 to 3.0 cm, and in 70% of those 3.1 to 5.0 cm in diameter (Table 1).

*Evaluation of HCC Efferent Veins*

Radiopaque media infused directly into tumors less than or equal to 5 cm in diameter allowed clear visualization of the pericapsular vascular network in 23 resected specimens. In 17 (74%) of 23 specimens, portal branches were fully visualized, while hepatic vein branches were not filled at all (Figure 2). In the other six cases (26%), both portal and hepatic vein branches were opacified, but the portal vein branch was always filled more predominantly than the hepatic vein branch. Thus, when the radiopaque media was infused into the tumors, the media flowed out in significantly higher rate into portal branches than into hepatic vein branches ($p<0.05$). In 3 minute HCCs out of 23 with an incomplete capsule, radiopaque media also drained directly into the surrounding noncancerous sinusoids at the site without a capsule and into portal branches at the portion with a capsule (Table 2). In the 23 resected specimens, 16 were histologically cirrhotic and 7 were noncirrhotic in the noncancerous liver. In the 16 tumors with cirrhotic liver 14 (87.5%) had visualization of only portal vein branches and 2 (12.5%) showed opacification of both the portal and hepatic vein branches, while in the 7 tumors with noncirrhotic liver 3 (42.8%) showed visualization of only portal vein branches and 4 (57.1%) revealed opacification of both the portal and hepatic vein branches.

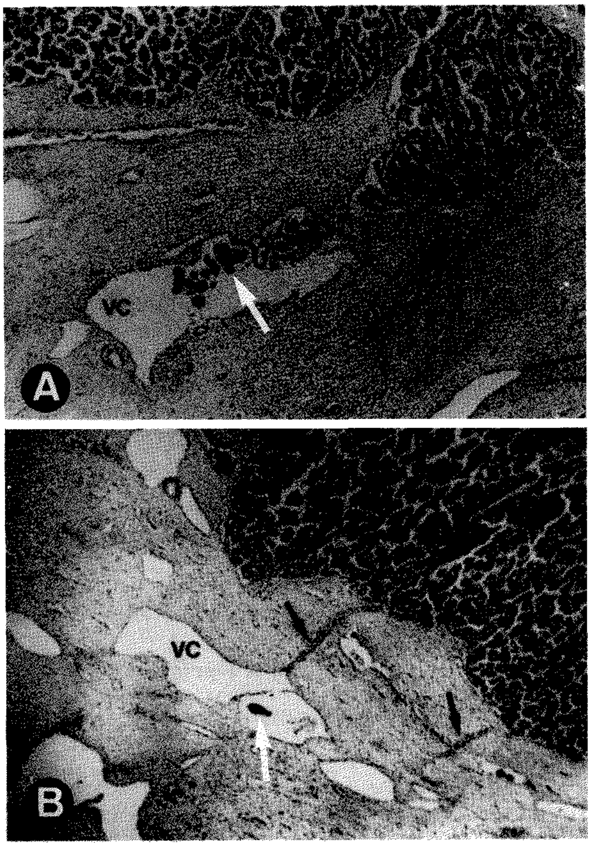

**Figure 1** (A) Microscopic finding of hepatocellular carcinoma invading into vascular channels (VC) of the tumor capsule. Tumor thrombi (white arrow) in the vascular channel of the tumor capsule are clearly identified (B). Fine communicating vessels (black arrow) between the vascular channels in the capsule and the blood space in the tumor also are observed (H&E stain; magnification ×100).

Direct portal infusion of the contrast media in the 7 resected specimens clearly demonstrated the vascular network of the capsule and abundant portal branches surrounding the tumors; however, radiopaque media did not enter the tumor in all the cases (Figure 3).

Siliconized rubber was infused into the tumor in three cases, and the interior of the tumor and the portal system around the tumor were visualized. Many portal branches around the tumor capsule penetrated directly into the interior of the tumor via fine communicating vessels (Figure 4).

The portal system around the primary tumor was examined radiographically in 8 patients with unresectable HCC by infusing radiopaque media directly into

**Table 1** Tumor size and degree of tumor invasion.

| Tumor size (cm) | | Histopathology of tumor spread: Capsular invasion | Extracapsular invasion | Vascular* invasion | Intrahepatic* metastasis |
|---|---|---|---|---|---|
| 0–2.0 | 33 cases | 32/33 (97.0%) | 20/33 (60.6%) | 16/33 (48.5%) | 12/33 (36.4%) |
| 2.1–3.0 | 93 cases | 92/93 (99.0%) | 71/93 (76.3%) | 55/93 (59.1%) | 46/93 (49.5%) |
| 3.1–5.0 | 105 cases | 105/105 (100.0%) | 86/105[a] (81.9%) | 77/105[b] (73.3%) | 74/105[b] (70.5%) |
| Total | 231 cases | 229/231 (99.1%) | 177/231 (76.6%) | 148/231 (64.1%) | 132/231 (57.1%) |

[a] $p < 0.05$ compared to 0–2.0 cm tumors
[b] $p < 0.05$ compared to 0–2.0 and 2.1–3.0 cm tumors
*Correlation between vascular invasion and intrahepatic metastasis (correlation coefficient = 0.998, $p < 0.05$

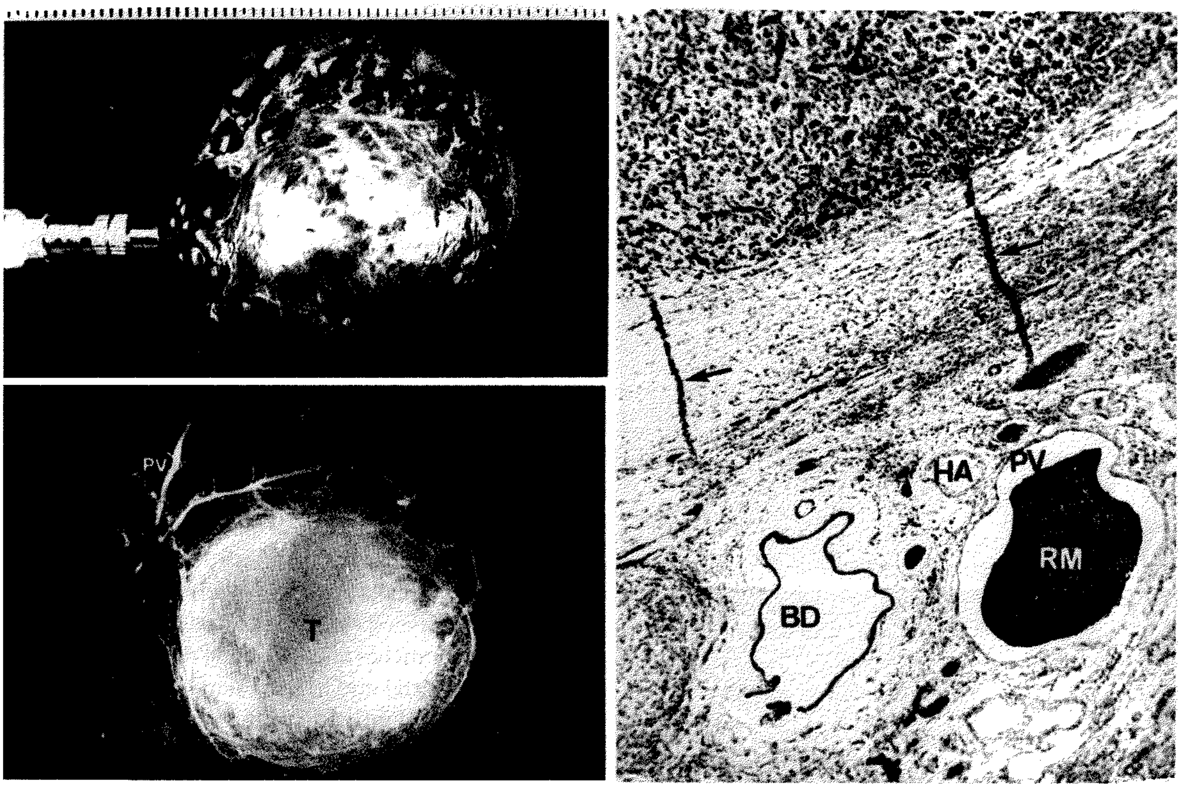

**Figure 2** Radiopaque media (RM) injected directly into a small hepaticellular nodule in the resected liver (upper left) demonstrated communication between the vascular channels in the tumor capsule and the bloodspace in the tumor (T) via fine vessels (lower left, ×100). The portal vein (PV) was clearly visualized by radiography and the radiopaque media was identified in the portal vein and the communicating vessel (arrows) on histologic examination (right, ×100).
(BD: Bile duct; HA: Hepatic artery)

the tumor by the percutaneous transhepatic route (Figure 5). The portal venous branches around the tumor was fully opacified, while the hepatic vein was opacified only faintly.

### DISCUSSION

Autopsy-based studies of HCC generally reveal many daughter (satellite) nodules; a single tumor is rare. Multiple tumor nodules in the liver have been assumed to be multicentric in origin since regenerative nodules in the setting of cirrhosis may frequently transform to HCC and because experimental HCC has been described as multicentric[6,7]. However, recent advances in imaging techniques have allowed detection of single, small, and often subclinical lesions. Early resection of such lesions has increased the long-term survival in several studies[1–5, 9–11, 13]. Daughter nodules could also be regarded as intrahepatic metastases since HCC has a high frequency of invasion into the portal venous system[1,5,9,11]. However, it remains difficult, even utilizing tests of DNA ploidy and clonicity to define accurately whether multiple tumors in HCCs are multicentric or metastatic lesions, because these tumors are characteristically heterogeneous.

Our study supports the notion that most daughter nodules are intrahepatic metastases. First, a definite primary lesion was present in all resected specimens, even when multiple tumors were present. Second, distinct daughter nodules were frequently found in the same portal branch area or an adjacent area. However, the most important finding was macroscopic thrombi in the portal vein in 18.3% of 393 cases and in the hepatic vein in only 3.1%. The reported incidence of tumor thrombi in intrahepatic vessels in HCC ranges from 47.6% to 91.8%[14,15]. In reports that described tumor thrombosis of both the portal and hepatic venous systems, the incidence in the portal system varied from 23.9% to 90.2% and in the hepatic venous system from 3.7% to 15.2%[5, 15–17]. Anyway the incidence of invasion of the portal system was always higher. In our series, the mode of tumor spread was analyzed among tumors $\leq$5 cm in diameter. These results suggest that serial tumor spread starts with intracapsular invasion, progresses to extracapsular

**Table 2** Roentgenographic findings in the 23 resected specimens in which radiopaque media was infused directly into the tumor ≤5 cm in diameter.

| *Case* | *Age/ Sex* | *Tumor size (cm)* | *Noncancerous condition* | *Visualization of portal vein* | *Visualization of hepatic vein* |
|---|---|---|---|---|---|
| 1. | 51 M | 5.0×4.5 | cirrhosis | # | – |
| 2. | 60 M | 4.5×3.0 | " | # | – |
| 3. | 60 M | 4.8×4.0 | chronic hepatitis | # | + |
| 4. | 50 M | 3.2×3.0 | cirrhosis | " | – |
| 5. | 48 F | 3.0×2.5 | " | " | – |
| 6. | 36 F | 5.0×4.5 | normal | " | + |
| 7. | 57 M | 2.5×2.3* | cirrhosis | " | – |
| 8. | 47 F | 4.8×4.7 | chronic hepatitis | " | – |
| 9. | 54 M | 5.0×4.6 | normal | " | + |
| 10. | 52 M | 3.0×2.5 | cirrhosis | " | – |
| 11. | 59 F | 3.7×3.5 | " | " | – |
| 12. | 52 M | 1.9×1.8* | cirrhosis | " | – |
| 13. | 51 F | 4.9×4.8 | chronic hepatitis | " | + |
| 14. | 48 M | 1.8×1.7* | cirrhosis | " | – |
| 15. | 49 M | 3.2×2.9 | " | " | – |
| 16. | 55 M | 4.6×4.0 | chronic hepatitis | " | – |
| 17. | 61 M | 2.0×1.9 | cirrhosis | " | – |
| 18. | 63 M | 2.9×2.6 | " | " | – |
| 19. | 46 F | 4.5×4.1 | " | " | + |
| 20. | 59 M | 3.3×3.1 | " | " | + |
| 21. | 66 M | 3.6×3.3 | " | " | – |
| 22. | 60 F | 5.0×4.8 | chronic hepatitis | " | – |
| 23. | 53 M | 2.5×2.3 | cirrhosis | # | – |
| The frequency of visualization of portal and hepatic veins | | | | 23/23 (100%)[a] | 6/23 (26.1%)[b] |

$p < 0.05$ (a vs b)
*The media drained directly into the surrounding noncancerous sinusoids at sites without a capsule

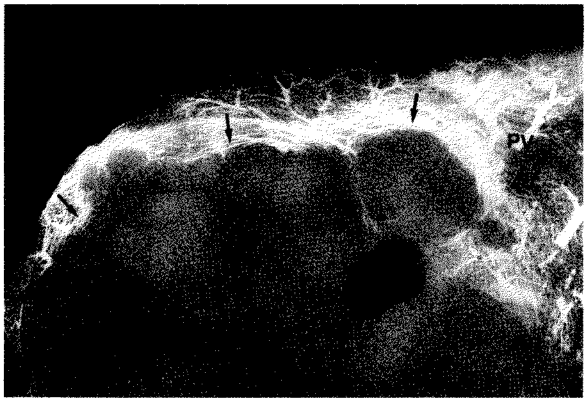

**Figure 3** Following injection of radiopaque media into the portal vein (PV) of resected specimen with hepatocellular carcinoma, a rich vascular network (arrows) was visualized around the tumor (T).

invasion, intravascular invasion, and finally to intrahepatic metastasis. Vascular invasion was observed in about one half and intrahepatic metastasis in one third of the tumors <2 cm, suggesting that HCC can invade the intrahepatic vascular system, especially the portal vein, at an early stage.

In our study, when radiopaque media was infused into the tumor, the media flowed immediately into portal vessels around the tumor with little resistance. The utilization of the portal vein as an efferent vessel was more marked when HCC was superimposed on cirrhosis. This finding was expected because of the impaired drainage by the hepatic vein in cirrhotic livers. This communication with the portal circulation in HCC could also be responsible for the early occurrence of intrahepatic metastasis in cirrhotics. Nakashima[18] has proposed that tumor thrombosis of the portal vein occurs at an early stage because the portal vein may act as the primary efferent vessel for HCC.

One possible objection may be that this study only proves that there were communications between the portal branches and the tumor, but cannot show that the portal vein serves as an efferent vessel. However, when contrast medium was injected into the tumor using a low injection pressure, contrast media entered the tumor with little resistance and flowed immediately out into the portal branches, suggesting that the contrast media was injected into vessels. Arteriography of HCCs generally shows hypervascularity.

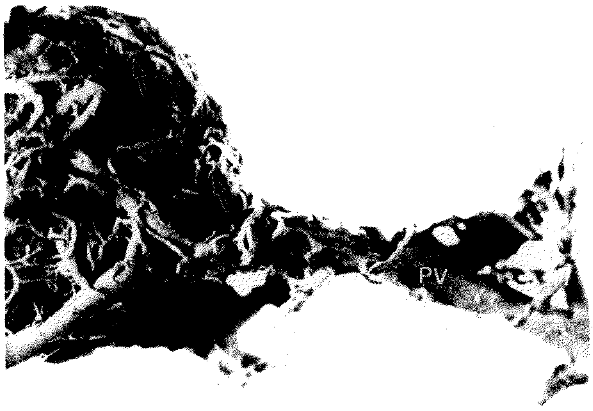

**Figure 4** A mold produced by direct infusion of siliconized rubber into an encapsulated hepatocellular carcinoma measuring 4 × 5 cm. The vascular network around the tumor (arrows) and the portal venous system (PV) is shown.

Abundant flow from the hepatic artery feeds the tumor, and arterial blood flows through the blood space into the tumor. Therefore, the pressure of blood flow into the tumor is much higher than that of the portal vein. Moreover, direct portal infusion of contrast media did not enter the tumor, although the portal area surrounding the tumor was fully opacified. Therefore we propose that the portal venous flow cannot enter the tumor, but serves only as an efferent vessel, and that the arterial vessels ordinarily serve as afferent vessels. The current study is the first in which efferent vessels in HCC were identified by direct infusion of radiopaque material into the tumor.

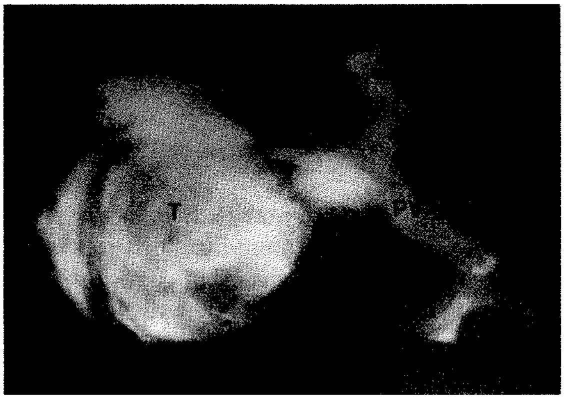

**Figure 5** Following injection of radiopaque media into a hepatocellular carcinoma (T) by the ultrasound guided percutaneous transhepatic puncture technique, the portal drainage system (PV) was visualized.

Our data suggests that efferent vessels penetrating the tumor capsule are the path of least resistance for tumor escape. We showed that tumor cells can invade efferent vessels, were engorged in the vascular cavity, and extend beyond the capsule to branches of the portal vein. This may be the major mechanism for the formation of tumor thrombi within the portal vein. It is also hypothesized that tumor fragments may disseminate via portal vein branches to form peripheral intrahepatic metastatic lesions. Tumor thrombi generally are fed by an artery of the primary tumor[15,18]. However, when thrombi of tumor fragments detach from the tumor and flow into the portal blood stream, they can survive in the portal blood. Then, they adhear to the portal vein wall and obtain their blood supply from the surrounding arterioles. Thus, the portal vein acts as a viable conduit for drainage of the blood feeding the tumor and even for tumor infiltration and spread. (Figure 6).

Our data demonstrates the presence of an arterio-portal (A-P) shunt in all HCCs, even in subclinical tumors and tumors without portal vein thrombosis. However, an A-P shunt is not detectable by conventional angiography in cases with a minor or no tumor thrombosis in the portal vein. But it can be often detectable in cases with a macroscoptic tumor thrombus. In this series, all the cases with an A-P shunt

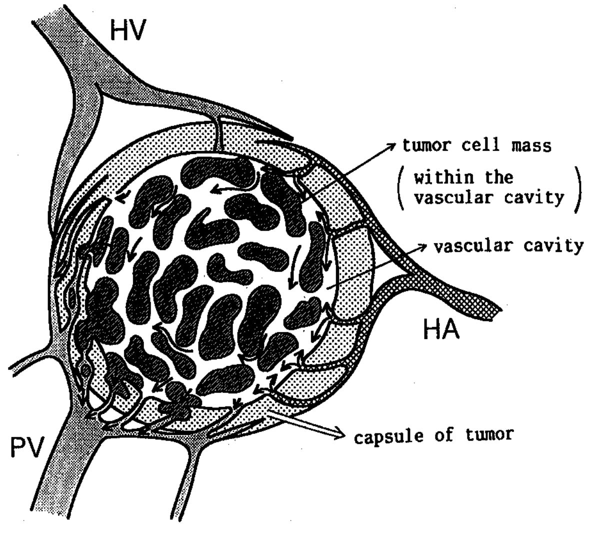

**Figure 6** Schematic drawing the vascular architecture in HCC. The hepatic artery is the feeding vessel, and the portal vein serves mainly as an efferent vessel. The efferent vessels penetrating the capsule are the path with least resistance for tumor infiltration or expansion. Tumor cells invade efferent vessels by budding, extension to the vascular cavity and extend beyond the capsule to the portal vein branches. (HA: Hepatic artery; PV: Portal vein; HV: Hepatic vein)

demonstrated by angiography had tumor thrombi in the major portal venous branches. When the tumor involves the portal vein, the arterial blood in the tumor naturally follows a pressure gradient and flows into the portal vein from the site of portal vein thrombosis. Therefore, A-P shunts may indicate the presence of tumor thrombi in the portal vein.

In summary, our findings suggest that the portal vein serves as an efferent vessel in HCC, rather than a supply vessel as suggested by others[19]. We hypothesize that this relationship accounts for the high frequency of portal vein thrombosis and intrahepatic metastasis in HCC.

### ACKNOWLEDGEMENTS

The authors thank Miss Keiko Mitani and Miss Akemi Sagae for manuscript preparation and their expert technical assistance.

### REFERENCE

1. Okamoto, E., Yamanaka, N., Toyosaka, A., Tanaka, N. and Yabuki, K. (1987) Current status of hepatic resection in the treatment of hepatocellular carcinoma. In *Neoplasms of the Liver*, edited by K. Okuda and K.G. Ishak, pp. 353–365. Tokyo: Springer-Verlag.
2. Yamada, R., Sato, M., Kawabata, M., Nakamura, K. and Takashima, S. (1983) Hepatic artery embolization in 120 patients with unresectable hepatoma. *Radiology*, **148**: 397–401.
3. Nakamura, H., Hashimoto, T., Oi, H. and Sawada, S. (1989) Transcatheter oily chemoembolization of hepatocellular carcinoma. *Radiology*, **170**: 783–786.
4. Makuuchi, M., Hasegawa, H. and Yamazaki, S. (1985) Ultrasonically guided subsegnebtectomy. *Surg. Gynecol. Obstet.*, **161**: 346–350.
5. Yamasaki, S., Hasegawa, H. and Makuuchi, M. (1981) Clinicopathologic observation of minute liver cancer and the new methods of hepatectomy; Analysis of 27 resected cases (in Japanese with English abstract). *Kanzo* (*Acta Hepatologica Japonica*), **22**: 1714-1724.
6. Anthony, P.P. (1973) Primary carcinoma of the liver: A study of 282 cases in Ugandan Africans. *J. Pathol.*, **110**: 37–48.
7. Foster, J.H. and Berman, M.M. (1977) Primary epithelial cancer in adults. In *Solid Liver Tumors*, edited by J.H. Foster and M.M. Berman, pp. 62–104. Philadelphia: W.B. Saunders.
8. Fortner, J.G., Maclean, B.J., Kim, D.K., Howland, W.S., Turnbull, A.D., Goldiner, P., Carlon, G. and Beattle, E.J. Jr. (1981) The seventies evolution in liver surgery for cancer. *Cancer*, **47**: 2162–2166.
9. Kishi, K., Shikata, T., Hirohashi, S., Hasegawa, H., Yamazaki, S. and Makuuchi, M. (1983) Hepatocellular carcinoma: A clinical and pathologic analysis of 57 hepatectomy cases. *Cancer*, **51**: 542–548.
10. Okuda, K. and Nakashima, T. (1981) Hepatocellular carcinoma: A review of the recent studies and development. In *Progress in liver diseases*, edited by H. Popper and F. Schaffner, vol. IV, pp. 639–650. New York: Grune and Stratton.
11. Okamoto, E., Tanaka, N., Yamanaka, N. and Toyosaka, A. (1984) Results of surgical treatments of primary hepatocellular carcinoma: some aspects to improve long-term survival. *World J. Surg.*, **8**: 360–366.
12. Tang, Z.Y., Ying, Y.Y. and Gu, T.J. (1982) Hepatocellular carcinoma: Changing concepts in recent years. In *Progress in liver diseases*, edited by H. Popper and F. Schaffner, vol.VII, pp. 637–647. New York: Grune and Stratton.
13. Wakasa, K., Sakurai, M., Okumura, J. and Kuroda, C. (1985) Pathological study of small hepatocellular carcinoma: Frequency of their invasion. *Virchows Arch. Pathol. Ahat.*, **407**: 259–270.
14. Patton, R.B. and Horn, R.C. Jr. (1964) Primary liver carcinoma: Autopsy study of 60 cases. *Cancer*, **17**: 757–768.
15. Kuwao, S. (1979) Histopathological studies on primary carcinoma of liver. Tumor thrombosis in the intrahepatic vascular system. *Acta Hepatologica Japonica*, **20**: 828–838.
16. Gustafson, E.G. (1937) An analysis of 62 cases of primary carcinoma of the liver based on 24,400 necropsies at Bellevue Hospital. *Ann. Intern. Med.*, **11**: 889–900.
17. Edmondson, H.A. and Steiner, P.E. (1954) Primary carcinoma of the liver. A study of 100 cases among 48,900 necropsies. *Cancer*, **7**: 462–503.
18. Nakashima, T. (1976) Vascular changes and hemodynamics in hepatocellular carcinoma. In *Hepatocellular carcinoma*, edited by K. Okuda and R.L. Peters, pp. 169–203. New York: John Wiley and Sons.
19. Honjo, I., Suzuki, T., Ozawa, K., Takasan, H., Kitamura, O. and Ishikawa, T. (1975) Ligation of a branch of the portal vein for carcinoma of the liver. *Am. J. Surg.*, **130**: 296–302.

### COMMENTARY

Hepatocellular carcinoma (HCC) accounts for more than 1 million deaths each year worldwide[1]. This tumour has two very interesting features which make the prognosis extremely poor. First, the operability rate is low, and second, intrahepatic recurrence is high after a successful hepatic resection.

Around 80 to 90 per cent of patients with HCC have inoperable tumours at the time of clinical presentation[2]. This is related to the relative lack of symptoms of the tumour until at an advanced stage of the disease, as well as the high frequency of portal vein invasion and multiple intrahepatic metastases. Resectional surgery is hazardous, and sometimes cannot be applied, because of the presence of chronic liver damage resulting from hepatitis B or C, alcoholism, and cirrhosis in more than three quarters of patients with HCC[3]. The use of screening employing serum alpha fetoprotein and ultrasonography in high risk patients has resulted in the detection of small asymptomatic tumours and improved on the operability rate and the long term survival rate.

In spite of the advances in chemotherapy, tumour irradiation, alcohol injection, cryotherapy and transarterial catheter embolisation, hepatic resection is the only procedure which offers the hope of cure.

Survival after liver resection is mainly affected by two factors: the underlying liver disease and recurrence of the tumour[4]. Poor liver function is predictive of late mortality, and more than half of the patients in Child's grade B and C died of progression of their liver disease within one year without tumour recurrence[5]. In patients with good liver function, the long term result of resection of HCC is affected by the high rate of intrahepatic recurrence, and only about 25 per cent of patients survive to 8 years[6]. However, very high cumulative intrahepatic recurrence reaching 100% at 5 years has also been reported[4].

The present study by Toyosaka and his associates sheds some light to the high incidences of tumour invasion of the portal vein and the intrahepatic recurrences after hepatic resection for HCC. These authors have demonstrated very nicely that the mode of HCC spread progresses from invasion of the tumour capsule, to extracapsular invasion, to vascular invasion and then to intrahepatic metastasis. Their findings, also suggest that intrahepatic invasion by HCC may occur through the portal vein as an efferent tumour vessel. The fact that intrahepatic metastasis was present in 57.1% of tumour <5 cm in this study suggests that limited liver resection for even small HCC may not be adequate and that adjuvant therapy after "curative" liver resection for HCC should be evaluated by randomised studies.

W.Y. Lau
A.K.C. Li
Department of Surgery
The Chinese University of Hong Kong
Prince of Wales Hospital
Shatin, New Territories
Hong Kong

### REFERENCES

1. Ezaki, T. (1992) Hepatocellular Carcinoma. BMJ **304**: 196–7.
2. Shiu, W. Dewar, G. Leung, N. *et al.* (1990) Hepatocellular carcinoma in Hong Kong: A clinical study on 340 cases. *Oncology*, **47**: 241–5.
3. McMaster, P., Mirza, D. and Harrison J.D. (1993) Surgical options for primary hepatocellular carcinoma. *Br J Surg*, **80:** 1365–7.
4. Belghiti, J. (1991) Resection of hepatocellular carcinoma complicating cirrhosis. *Br J Surg*, **78**: 257–8.
5. Franco, D., Capussotti, L. Smadja, C. *et al.* (1990) Resection of hepatocellular carcinomas. Results in 72 European patients with cirrhosis. *Gastroenterology*, **98**: 733–8.
6. Nagasue N. Kohno H. Chang Y.C. *et al.* (1993) Liver resection for hepatocellular carcinoma: results of 229 consecutive patients during 11 years. *Ann Surg*, **217**: 375–84.

